# Multidrug-resistant *Mycobacterium tuberculosis*: a report of cosmopolitan microbial migration and an analysis of best management practices

**DOI:** 10.1186/s12879-020-05381-0

**Published:** 2020-09-17

**Authors:** Oana Joean, Thea Thiele, Katharina Schütz, Nicolaus Schwerk, Ludwig Sedlacek, Barbara Kalsdorf, Ulrich Baumann, Matthias Stoll

**Affiliations:** 1 Department for Rheumatology and Clinical Immunology, Hannover Medical School, Hannover, Germany; 2 Department of Respiratory Medicine, Hannover Medical School, Carl-Neuberg-Str 1, 30625 Hannover, Germany; 3 Department of Pediatric Pneumology, Allergy and Neonatology, Hannover Medical School, Hannover, Germany; 4 Institute for Medical Microbiology and Hospital Epidemiology, Hannover Medical School, Hannover, Germany; 5 Division of Clinical Infectious Diseases, Research Center Borstel, Borstel, Germany; 6 German Center for Infection Research, Clinical Tuberculosis Center, Borstel, Germany

**Keywords:** Tuberculosis, Molecular epidemiology, Case series

## Abstract

**Background:**

Tuberculosis (TB) control is a primary global health priority but the goal to eliminate TB is being threatened by the increase in incidence of multidrug-resistant tuberculosis (MDR-TB). With this series of seven MDR-TB cases in migrant patients with identical *Mycobacterium tuberculosis* strains we aim to illustrate the challenges encountered during therapy and follow-up: language barriers, access to care for migrant patients, depression due to isolation, adverse reactions to the treatment, management of pediatric TB, further contact tracing. We also discuss best practices for the management of complex MDR-TB cases in settings with low overall TB incidence focusing on modern diagnostic assays and an individualized and an interdisciplinary therapeutic approach.

**Methods:**

We describe a case series of seven consecutively diagnosed MDR-TB patients, six of them treated at our tertiary care hospital between May 2018 and March 2020. Epidemiologic data was gained by semi-structured patient interviews and reconstruction of the migration route. The origin of the cluster was confirmed by genotyping of the TB-strains.

**Results:**

Six related patients were diagnosed with pulmonary MDR-TB between May and August 2018. All had a positive Interferon-Gamma-Release Assay (IGRA), in five patients sputum microscopy was positive for acid-fast bacilli (AFB). The genetic and phenotypical drug susceptibility test did not match with MDR-TB strains from an East-African origin. The index patient was identified through genetical fingerprinting. By changing the therapy to a modern MDR-TB regime and using an interdisciplinary and culture-sensitive approach, all patients improved clinically and radiologically.

**Conclusion:**

Human migration plays an important role for the global spread of MDR-TB in low incidence countries. Early case detection and adequate treatment are key to prevention of outbreaks. Especially language barriers and complex migration routes make genotyping of TB-strains a crucial tool to identify cases clusters, the potential index patient and transmission dynamics. We are fortunate enough to experience times in which new TB-antibiotics were made available and in which molecular assays revolutionized TB-diagnostics. We need to take advantage of that and develop personalized therapies for patients suffering from drug resistant TB.

## Background

Tuberculosis (TB) is the leading cause of death from a single infectious agent [1]. The incidence of drug-resistant TB has increased steadily in the past years, threatening the goal to end the TB pandemic [1]. In patients with or multidrug-resistant (MDR-TB), defined as an infection by a *Mycobacterium tuberculosis* complex (MTBC) strain showing resistance to at least rifampicin and isoniazid treatment is expensive, unacceptably long, poorly tolerated and has a low success rate [2, 3]. The global spread of MTBC strains has been closely linked to human migration [4]. Armed conflicts, natural disasters and poverty have led to unprecedented forced displacement. In 2018, 70.8 million people, half of them children, were forced to flee their homes [5]. Newly notified TB case numbers are comparably low in North American and Western European nations and typically range below 25 per 100,000 people [6]. In these settings, previous TB treatment and a foreign-born status are the strongest risk factors for MDR-TB [7, 8]. Although genome-based molecular surveillance has significantly improved the assessment of TB transmission dynamics, it is still only irregularly used. Frequent relocation as well as language and cultural barriers pose significant challenges for contact tracing in migrant populations [9–11].

Based on a series of seven migrant patients with MDR-TB, we discuss best practices for the management of complex MDR-TB cases in settings with low overall TB incidence focusing on the state-of-the-art in diagnostics and personalized treatment, transmission, access to healthcare, social and language barriers.

## Methods

### Epidemiological methods/contact tracing

After diagnosing MDR-TB in two migrant patients (mother and child) that were infected with a *M. tuberculosis* strain resistant to isoniazid (INH), rifampicin (RMP), ethambutol (EMB), pyrazinamide (PZA), ethionamid (ETO) and moxifloxacin, a case series investigation was initiated. Contact tracing was pursued by semi-structured interviews in German, English and Arabic (Additional file 1). Contacts were considered persons that had had contact with the culture-confirmed cases during the symptomatic phase of TB disease before diagnosis. IGRA Tests, sputum analyses (smear, Tb-specific nucleic acid amplification test, culture) and chest X-rays were performed in all presumed contacts. The data were confirmed by genetic fingerprinting of TB in culture-positive contacts. In an attempt to identify the index-case, the semi-structured interviews focused on the exact migration route of each patient as well.

### Drug susceptibility testing

MTBC isolates were obtained by culturing primary specimens in both liquid (BACTEC MGIT 960, Becton Dickinson Diagnostic Systems, Sparks, US) and on solid media (Löwenstein-Jensen and Stonebrink media). Phenotypic drug susceptibility testing (DST) for all drugs except cycloserin (solid medium) was performed using the MGIT 960 system at critical concentrations as recommended by WHO [12]. For molecular DST the Genotype MTBDR*plus* and MTBDR*sl* assays (HAIN lifescience GmbH, Nehren, Germany) were performed according to manufacturer recommendations. Mutations within the pyrazinamidase gene *pnc*A were detected by amplification and DNA sequencing. Phenotypic DST of second-line antituberculous drugs were performed via the MGIT 960 system.

### Whole genome sequencing

For strain comparison and extended analysis of resistance associated mutations whole genome sequencing (WGS) was performed with Illumina Technology using NextSeq500 and Nextera XT library preparation kits as instructed by the manufacturer (Illumina, San Diego, CA, USA). Obtained reads were processed with the MTBseq pipeline calling variants with a minimum of 4 reads in both forward and reverse orientation, 4 reads calling the variant with at least a phred score of 20, and 75% variant frequency [13].

### Clinical data

The clinical characteristics presented were the routine clinical data collected in the course of treatment and follow-up. No additional tests or procedures were performed for the sole purpose of this study.

## Results

### Microbiological data and genome-based molecular surveillance of TB

Six patients from two related migrant families originating from Sudan were diagnosed with MDR-TB caused by a MTBC strain showing a complex pattern of phenotypic resistance and resistance associated mutations for isoniazide (INH), rifampicin (RMP), ethambutol (EMB), pyrazinamide (PZA), ethionamid (ETO) and low-dose fluorochinolones - a strain that had never been diagnosed in Germany in patients originating from East Africa (Table 1). By contact-tracing via semi-structured interviews (Additional file 1) that was verified by genetic fingerprinting of TB, we identified the probable index patient (patient 7). It was a 50-year-old Georgian migrant that had lived in the same refugee accommodation with the families for 7 days before being diagnosed with MDR-TB. Data on contact tracing performed on the index patient were not available.

**Table 1:** Resistance pattern of the MDR-TB strain

Drug	Phenotypical susceptibility testing	Associated mutations
Rifampicin/Rifabutin	Resistant	*rpoB* gene: mutation S531L
Isoniazid	Resistant	*katG* gene: mutation S315T
Isoniazid/Ethionamide	Resistant	inhA promotor: mutation T-8C
Ethambutol	Resistant	*embB* gene: mutation M306I
Pyrazinamide	Resistant	*pncA* gene: mutation T47A
Levofloxacin	Resistant (0,5 mg/l)	*gyrA* gene: wild type *gyrB* gene: mutation E540N
	Susceptible (1 mg/l)	
Moxifloxacin	Resistant (0,25 mg/l)	
	Susceptible (1 mg/l)	
Aminoglycosides	Susceptible	*rrs* gene: wild type *eis* gene: wild type
Cycloserine	Susceptible	
Para-aminosalicylic acid (PAS)	Susceptible	
Linezolid	Susceptible	
Bedaquiline	Susceptible	
Clofazimine	Susceptible	
Delamanid	Susceptible	

### Clinical and radiological findings

Three of the six patients were children: two infants and a 4-year old girl. The adults and the 4-year old girl had fled Sudan. Both infants (patients 2 and 6) were born in Germany and had been previously diagnosed with recurrent bronchitis unresponsive to symptomatic treatment but had never had an X-ray. None of them was infected with the human immunodeficiency virus.

**Fig. 1:**
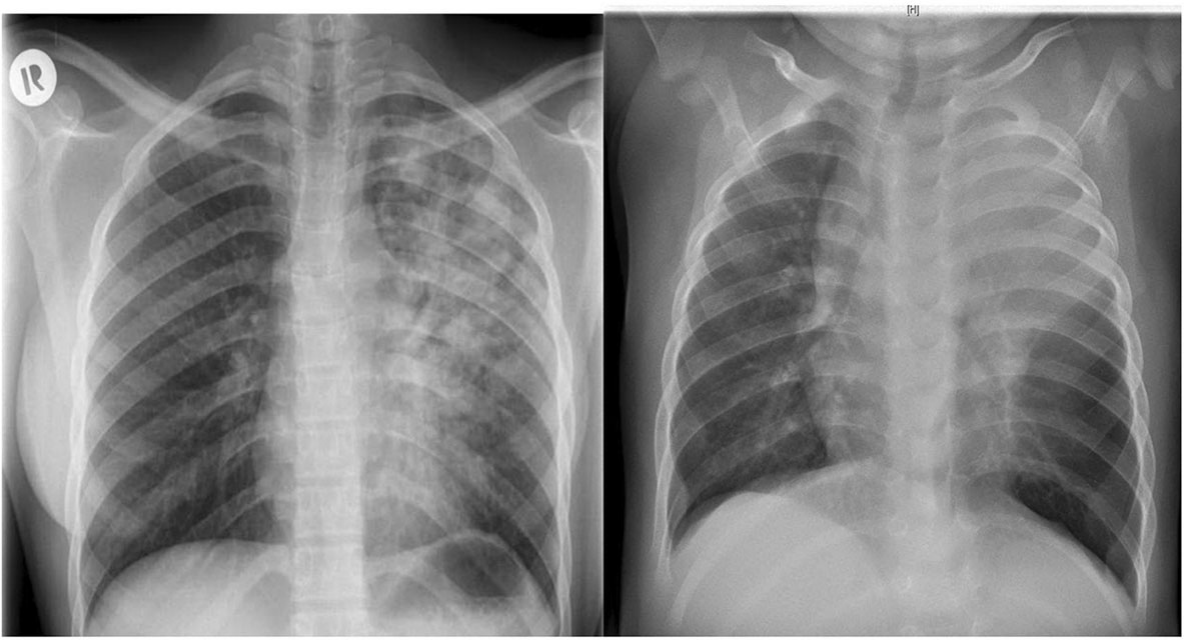
Chest X-ray studies showing extensive TB-lung involvement in both the adult (left) and the pediatric patient (right)

In April 2018 a 21-year old woman (patient 1) and her 6 months old son (patient 2), were diagnosed with pulmonary TB non-responsive to standard quadruple therapy (INH, RMP, EMB, PZA). In May 2018, both patients were transferred to our infectious disease department for further care, no genotyping had been performed beforehand. Patient 1 had experienced fever, loss of appetite, weight loss, cough and dyspnea since January 2018. At diagnosis, sputum microscopy was highly positive for acid-fast bacilli (AFB) and culture became positive for MTB within 6 days. Chest X-rays showed left-sided lung cavities (Fig. 1a). Her breastfed son (patient 2) showed disseminated bronchial involvement, bilateral mediastinal lymphadenopathy, fever, and failure to thrive. In the course of subsequent contact tracing investigations, both the 29-year old husband (patient 3) and the 4-year old daughter (patient 4), who were clinically asymptomatic, showed highly positive Interferon-gamma release assay (IGRA)-Tests and radiographic signs of TB including mediastinal lymphadenopathy and apical pulmonary noduli. While patient 3 was culture-positive for TB, all mycobacteriological investigations for patient 4 remained negative (Table 2).

**Table 2:** Patient characteristics

Patient	Sex	Age at diagnosis	Country of birth	Diagnosis	Disease mani-festation	AFB microscopy	TB culture from respiratory sample	IGRA
Patient 1	F	20 y	Sudan	05/2018	Pulmonary	+++	+	+
Patient 2	M	6 mo	Germany	05/2018	Pulmonary	+++	+	+
Patient 3	M	28 y	Sudan	08/2018	Pulmonary	(+)	+	+
Patient 4	F	3 y + 10 mo	Sudan	07/2018	Pulmonary	–	–	+
Patient 5	F	19 y	Sudan	06/2018	Pulmonary	(+)	+	+
Patient 6	M	6 mo	Germany	06/2018	Pulmonary	++	+	+
Patient 7 (index)	M	50 y	Georgia	06/2017	Pulmonary	+++	+	+

Legend: *AFB* Acid Fast Bacili, *F* female, *M* male, *y* years, *mo* months, *AFB* acid fast bacilli, *TB* Tuberculosis, *IGRA* Interferon-Gamma-Release Assay, AFB microscopy: +: 10–99 AFB/100 fields; ++: 1–9 AFB/field; +++: > 9/field

Two additional cases were identified within a closely related family: a 20- year old woman (patient 5) was asymptomatic but her sputum turned culture positive for *M. tuberculosis*. Chest CT scans revealed multiple granulomas in the apical lung segments and hilar lymphadenopathy. Her 6-months old son (patient 6) showed mediastinal lymphadenopathy with compression of the left main bronchus and an extensive left apical pulmonary infiltrate (Fig. 1b). *M. tuberculosis* was cultured from bronchoalveolar lavage. Disseminated TB was excluded in all children through abdominal ultrasound and lumbal puncture.

### Treatment strategy, side-effects and outcomes

Treatment was initiated for all adult patients based on genotypic and phenotypic drug sensitivity testing results and included bedaquiline, clofazimine, linezolid, terizidone, amikacin, 4-aminosalicylic acid (PAS), and meropenem in combination with amoxicilin/clavulanic acid. The children received clofazimine, linezolid, terizidone, amikacin, and PAS. Both treatment strategies were designed taking into consideration the current TB national guidelines and the drug sensitivity results available at the time of referral [14]. Laboratory screening (serum creatinine, electrolytes, liver enzymes, thyroid stimulating hormone, haemoglobin and white blood count), assessment for peripheral neuropathy, audiometry, and electrocardiograms were performed following the current recommendations to monitor adverse effects. All patients developed slight liver enzymes elevations, mild high-frequency hearing loss during long-term treatment with aminoglycosides, bone marrow suppression while receiving linezolid and a mild hypothyroidism presumably associated with PAS. All patients exposed to oral PAS showed moderate gastrointestinal distress including bloating and nausea aggravating malnutrition especially in patient 1. Four of the patients developed a QTc-prolongation up to 493 msec.

**Fig. 2:**
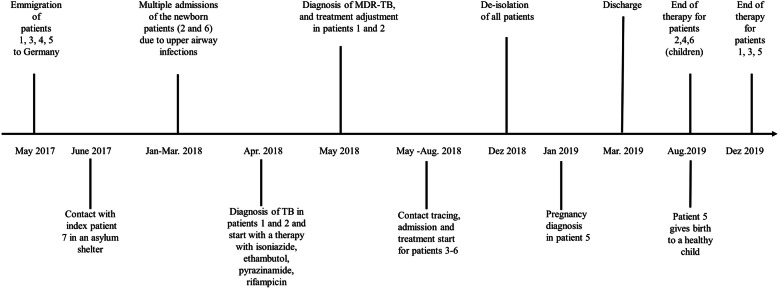
Timeline of the clinical case presentation

Cohort isolation – by families – was necessary up to 7 months until three consecutive sputa were microscopically negative for AFB and culture was negative. Patient 5 became pregnant about 5 months into the isolation period and, after an uneventful pregnancy despite extensive TB treatment, she gave birth to a healthy child. The patients were discharged with continuation therapy of bedaquiline, levofloxacin, terizidone, clofazimine, and PAS in the adult cases or levofloxacin, terizidone, clofazimine, and PAS in the infant cases. Meropenem was discontinued to simplify outpatient care, the other antibiotics were chosen based on final drug sensitivity results and on drug tolerability. They all completed the treatment with no signs of relapse (Fig. 2).

## Discussion

Gathering data, but also overcoming the social, cultural, diagnostic and therapeutic challenges involved a coordinated effort of public health officers, microbiologists, an interdisciplinary team of infectious disease’ practitioners and pediatricians, microbiologists, obstetricians, nurses, medical students and volunteers. We discuss challenges in the management of MDR-TB in a low incidence country and possible ways to overcome them.

### Rapid diagnosis of TB and molecular typing of MTBC strains

Rapid and accurate diagnosis is essential to prevent inadequate treatment of MDR-TB. TB diagnosis is based on clinical, radiological and microbiological features. However, clinical features can be heterogeneous and are, especially in children, less specific ranging from weight loss, cough and fever to wheezing or lethargy, possibly delaying the diagnosis in low-burden settings. In recent years, genome-based characterization of MTBC strains became a powerful tool for prospective surveillance and prediction of antimicrobial susceptibilities. In the setting of population displacement, language barriers and fragmented information related to contacts, genotyping has been used to infer the likelihood of transmission between cases [9–11]. The advent of extensive genetic DST enables targeted and more individualized treatment regimens [15].

Genotypic drug sensitivity testing (DST) was only performed after referral to our clinic, leading to an ineffective standard TB-therapy of the first two patients for weeks. In our cases it revealed identical TB-strains in all culture-positive patients and-by revealing the complex resistance pattern- it also enabled us to find the index patient. Phenotypic and genotypic DST was concordant for all drugs tested (Table 1).

Laboratories need to implement standardized algorithms for TB diagnosis: nucleic acid amplification tests endorsed by the World Health Organisation should be performed on at least one sputum sample per patient being evaluated for *MTBC strains*. Rapid drug susceptibility tests should be more accessible in primary care hospitals to provide comprehensive and efficient care. In future, whole genome sequencing could be the tool to detect a broad spectrum of mutations with one analysis [10, 16].

### Contact tracing and access to national healthcare systems for migrants with MDR-TB

Forced displacement, poor nutrition, the collapse of health service infrastructure, mental and physical stress are risk factors for refugees contracting infectious diseases, including TB [17]. In Germany, all asylum seekers above 16 (excluding pregnant women) undergo a compulsive TB screening via chest X-ray upon entering the country. The federal states organize reception and registration independently, including TB screening and documentation. Data transfer and storage was poorly standardized. Therefore good quality and reliable data concerning health monitoring of refugees and TB in particular in Germany are scarce [18].

As a result, only the adult male patients were screened for TB upon entering Germany. The X-rays were only available after intensive file studies and involving many health authorities but showed no signs of active TB upon entering Germany. No IGRA tests were performed.

**Fig. 3:**
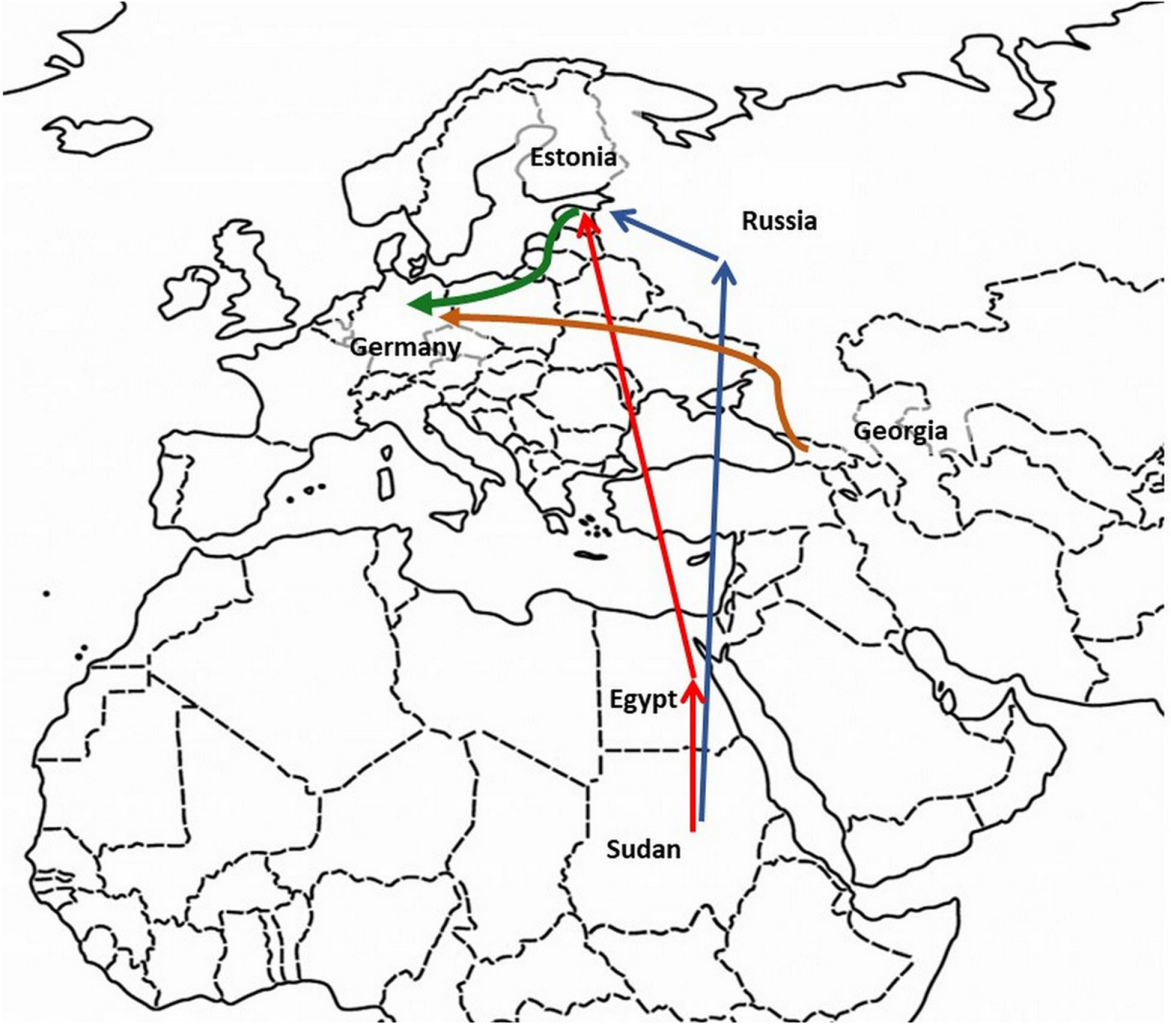
Travel routes of the cases (modified from VectorStock®, Expanded Licence ID 21681944, licensed to the corresponding author). Case 1, 4 and 5 travelled from Sudan via Egypt to Estonia (red line). Case 3 travelled from Sudan via Russia to Estonia (blue line). Cases 1, 3, 4 and 5 reunited in Estonia and further travelled to Germany (green line). Case 7 (index case) travelled from Georgia to Germany (orange line)

Our patients couldn’t remember much about the first medical screening and trying to recapitulate their migration route and their displacements within Germany necessitated numerous interviews. Before immigration to Germany the adults and the 4-year old girl were residents of Sudan. The male patients had travelled to Germany via Russia and Estonia in 2014, the female patients – 2 adults and one child- had travelled via Egypt to Estonia by airplane. They reunited with the male patients and continued their route to Germany by bus (Fig. 3). Both infants were born in Germany approximately 3 months after arriving there. Before travelling to Germany in May 2017, the families had lived in two flats in Estonia with no contact to other refugees. None of the patients remembered contact to a coughing or ill person during this period. They only remembered having lived with an ill middle-aged person for a couple of days in one of the first German refugee camps. This person could be identified as a 50-year-old Georgian refugee who was diagnosed with cavernous pulmonary MDR-TB briefly after having lived with our patients and who was successfully treated in another hospital in northern Germany. Through genetic fingerprinting of MTBC we identified this patient as the index case.

In Germany, access to health care services for asylum seekers is limited to emergencies, with exceptions being possible on a case-by-case basis, which leads to delays in the delivery of care [18, 19]. Consequently, access to long and expensive MDR-TB treatments for this population is poorly regulated. According to the Dublin Regulation, the first country of entry to the European Union is responsible for a person’s asylum claim. Five months after starting treatment of our first patients they faced the possibility of being sent to Estonia, the first European country they set foot on, putting the treatment continuation and therefore outcome in jeopardy. For this reason, although the patients were diagnosed with a life-threatening disease in one of the wealthiest countries in the world, there were serious uncertainties about whether the high therapy costs will be covered by the national health-care system.

Due to the national and international mobility of asylum seekers, a joint European solution of health information management would be desirable for the future.

### Personalized treatment of patients with MDR-TB

Almost 50 years after the introduction of rifampicin, three new TB-antibiotics (bedaquiline, delamanid, pretonamid) were lanced and a number of older and repurposed drugs became increasingly important for the treatment of MDR-TB [2, 20–25]. As drug-susceptibility testing is complemented by genotypic methods offering rapid information on drug resistance patterns, individualized MDR-TB regimens can be designed more rapidly - an approach we also used. However, MDR-TB regimes are poorly tolerated, lengthy and expensive. Because of the adverse effects up to 90% of patients need regimen changes [2, 3, 26]. Our patients did not make an exception so we needed to adapt the treatment plans to the severe adverse effects, for example the severe polyneuropathy leading to discontinuation of linezolid in the adult patients or the high frequency hearing loss occurring in all patients treated with amikacin. All patients receiving an MDR-TB treatment should undergo systematic clinical and laboratory assessment to detect and allow rapid management of drug toxicity and adverse events [27, 28]. Standardized data should be systematically collected and reported to inform future policies on MDR-TB treatment strategies.

Aiming to provide family-centered care, we decided not to separate the children from their parents. Therefore, the first family was admitted to the adult infectious disease ward and the last two cases were treated on the pediatric ward. Infectious disease practitioners worked close with pediatricians and therapeutic decisions were always made in this interdisciplinary team.

### Pediatric TB

Although it is estimated that up to 32.000 children develop MDR-TB every year, little is known about optimal treatment strategies [29, 30]. Diagnosing TB in children is more strenuous than in adults because of difficulties in collecting respiratory samples and because children often have paucibacillary (AFB smear- and culture-negative) disease [31]. Moreover, care for pediatric MDR-TB patients is hampered by a lack of child-friendly formulations for second-line therapeutic regimens. Secondly, children have a wide spectrum of TB manifestations and the clinical features are rarely straight-forward [29, 31]. Both infants (patient 2 and 6) had been seen by general practitioners and pediatricians due to recurrent airway distress and failure to thrive but were only diagnosed after months of delay. Therefore, even in low-burden settings TB should be considered in the differential diagnosis and molecular diagnosis of TB should be implemented early on.

### TB and pregnancy

The immunological changes associated with pregnancy render pregnant women more vulnerable to TB [32, 33]. The unawareness of TB in Germany and the fear of X-ray studies during pregnancy delays the diagnosis and treatment in this population. We assume that both women were infected while being pregnant but were only diagnosed months afterwards.

Both young families were explained the need for contraception measures during the treatment, only one of the families opted for an intrauterine device. Five months into the isolation period of patient 5 we diagnosed an early pregnancy (gestation age 11 weeks). This raised additional ethical and pharmacological issues since we now needed to balance the possible teratogenic risks of the therapy against the risk of insufficient treatment. At this point patient 5 was treated with levofloxacin, bedaquilin, terizidone, clofazimine and PAS. There is little data on the safety and efficacy of these drugs during pregnancy and most data relating MDR-TB pregnant women were gathered from case reports and case series [34–36]. The expecting parents were counselled by gynecologists, infectious disease specialists, psychologists and pharmacologists and they decided not to interrupt the pregnancy; the treatment was also continued unchanged. After an uneventful pregnancy patient 5 gave birth at full term to a healthy child. Since the mother had been smear negative for months now, she and the newborn weren’t separated. The newborn didn’t receive any TB prophylaxis after birth and- respecting the parents’ wishes and their cultural background- it was breastfed.

TB management in pregnancy is a multidisciplinary approach. Moreover, family planning counselling should be an integral part of the therapy and follow-up strategies for TB patients of child-bearing age.

### Language and cultural barriers

Language and cultural barriers hindered adherence and limited the quality of information provided to the patients, since it often lacked detail. Language barriers also diminished the accuracy in reporting side-effects and it was challenging to verify the patients’ understanding. Depression due to isolation also posed difficulties. The new pregnancy underscored the differences in therapeutic goals and disease understanding between medical personnel and patients: the young couple was explained the need for contraception measures during the treatment and-since they refused following medical advice- we didn’t approach the issue further.

We addressed all the above issues with the support of translators from bilingual colleagues and volunteers from the migrant community. Professional translators and available psychological support dealing with pre-migration trauma and post-resettlement stress among refugees would be desirable and would have probably improved adherence in our patients.

## Conclusion

Tuberculosis control is a primary global health priority but the goal to eliminate TB is being threatened by the increase in incidence of MDR-TB cases [1, 23]. Travel and migration are phenomena as old as humankind and- since they have reached unprecedented levels in the past years – they are due to also speed up the geographical spread of multidrug resistant strains. This case series demonstrates the special challenges of MDR-TB in a low incidence country with a decentralized health care system. We are fortunate enough to experience times in which new TB-antibiotics were made available and in which molecular assays revolutionized TB-diagnostics. We need to take advantage of that, establish early molecular DST and develop personalized therapies for patients suffering from drug resistant TB. Hopefully, in the future access to routine next generation sequencing of specimens from every TB patient will enable us to improve a comprehensive surveillance, detect TB case clusters and trace transmission patterns faster and more efficiently. Furthermore, integrating prevention and relief strategies for the physical, psychological and social distress brought by TB is both medically and morally essential.

## Supplementary information

**Additional file 1.** Semi-structured interview concerning the migration routes, diagnosis of TB and contact tracing.

**Additional file 2.** Expanded license agreement and copy-right for Figure 3, modified from VectorStock® and licensed to the corresponding author.

## Data Availability

Data will be made available by the corresponding author upon reasonable request.

## Abbreviations

TB: Tuberculosis
MDR-TB: Multidrug-resistant tuberculosis
MTBC: *Mycobacterium tuberculosis* complex
INH: Isoniazide
RMP: Rifampicin
EMB: Ethambutol
PZA: Pyrazinamide
ETO: Ethionamid
DST: Drug susceptibility testing
AFB: Acid-fast bacilli
IGRA: Interferon-gamma release assay
PAS: 4-aminosalicylic acid
