# Supplementary material for: Multidrug-resistant Mycobacterium tuberculosis: a report of cosmopolitan microbial migration and an analysis of best management practices
Source: BMC Infect Dis. 2020 Sep 17;20:678. doi: 10.1186/s12879-020-05381-0 (PMC7499973; doi:10.1186/s12879-020-05381-0)
Supplement: Supplementary file 1 — Additional file 1. Semi-structured interview concerning the migration routes, diagnosis of TB and contact tracing. [file 12879_2020_5381_MOESM1_ESM.pdf]

Introductory questions

Name:

Sex:

Date and place of birth:

Nationality:

Please tell us which country and town did you leave to come to Germany and when was that?

Open questions:

Migration route to Germany:

Please explain how you arrived to Germany/what migration route to Germany you took

Prompts: means of transportation, countries transited, countries and cities you resided on your way to Germany, accommodation, crossed borders, persons travelling together

Displacement within Germany

Please explain where you lived so far in Germany

Prompts: places you lived in and for how long, types of accommodation, persons you shared accommodation with

Medical screening upon arriving to Germany

Do you remember whether you received a medical examination upon entering Germany and, if so, what it entailed?

Prompts: physical examination, X-ray, blood samples

Knowledge about tuberculosis before migrating

Do you know what tuberculosis is and-if so-have you or any persons in your environment ever had tuberculosis?

Prompts: long-term TB antibiotic intake, cough, fever, loss-of-weight before leaving the country of birth, contact to sick persons before leaving the country of birth

First TB symptoms and time-to-diagnosis

Please explain when you or your child started feeling ill, what symptoms you experienced and what doctors you saw until the diagnosis of TB

Prompts: fever, cough, loss of appetite, weight loss, medical consultations (MD, date of consultation) and consequence thereof, prescribed medications prior to the diagnosis of TB

Contact tracing

Have you had contact to ill persons on your route to Germany or in Germany?

With whom have you been in contact since starting feeling ill?

Prompts: time and type of contact, symptoms the contacts experienced, treatments the contacts might have received
