# Supplementary material for: Multidrug-resistant Mycobacterium tuberculosis: a report of cosmopolitan microbial migration and an analysis of best management practices
Source: BMC Infect Dis. 2020 Sep 17;20:678. doi: 10.1186/s12879-020-05381-0 (PMC7499973; doi:10.1186/s12879-020-05381-0)
Supplement: Supplementary file 2 — Additional file 2. Expanded license agreement and copy-right for Figure 3, modified from VectorStock® and licensed to the corresponding author. [file 12879_2020_5381_MOESM2_ESM.pdf]

VectorStock®

IMAGE PURCHASES

VectorStock.com
sales@vectorstock.com

Licensed to - Oana Joean

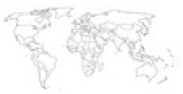

ID	NAME	
21681944	Contour world map black and white colors vector	
**LICENSE**	**PRICE**	**DATE**
Expanded License	0 Credit	May 6, 2020

View - EULA Standard | EULA Editorial | EULA Expanded

Re: Expanded License VectorStock

VectorStock Administration [admin@vectorstock.com]
Gesendet: Mittwoch, 6. Mai 2020 22:35
An: Joean, Oana Dr.

Dear Oana,

Yes you can use our images in this way with the Expanded License,

Best Regards / Admin Team

VectorStock®

WATCH TWEET PIN SHARE

WWW.VECTORSTOCK.COM \ THE WORLDS PREMIER ONLINE VECTOR IMAGE AGENCY

VECTORSTOCK MEDIA LIMITED \ PO BOX 68647 \ NEWTON \ AUCKLAND \ NEW ZEALAND 1145 \

CAUTION : this electronic e-mail message and any attached files contain information intended for exclusive use of the individual or entity to whom it is addressed and it may contain information that is proprietary, privileged, confidential and/or exempt from disclosure under international law. If you are not the intended recipient, you are hereby notified that any viewing, copying, disclosure or distribution of this information may be subject to legal restriction or sanction. Please notify the sender, by electronic mail of any unintended recipients and delete the original message without making any copies. The views or information expressed in this message are those of the author and may not be those of Vectorstock Media Limited, the directors or the website **www.vectorstock.com**

On 7/05/2020, at 7:33 AM, <Joean.Oana@mh-hannover.de> <Joean.Oana@mh-hannover.de> wrote:

Good evening!

I recently purchased a vector image with an extended license from VectorStock. Am I allowed to publish parts of this image in a non-vectorial form for a scientific paper under a CC BY open access license?

Best regards,
Oana Joean

Search... All Vectors Free Vectors Categories Buy Images

Account

USER
Oana

Credits 30 CR
My Purchases

Account
History
Transactions

COLLECTIONS

Cart 0
Galleries 0
Likes 0
Following Artists 0

Log Out

FAQ

Frequently Asked Questions

Members Artists

VectorStock

- What is VectorStock®?
- VectorStock® Website Terms of Use
- What can I use VectorStock® content for?
- About Us
- Contact Us
- VectorStock® disclaimer
- VectorStock® Take Down Policy

Vectors

Licensing

- Why are the files at such a low cost?
- License Types - Standard
- License Types - Expanded
- I need to upgrade a file to an Expanded License
- Free Vector usage with image Credit/Attribution
- VectorStock® Membership and Content License Agreement
- Free Vector License

Pay-per-Image

Credits

Subscriptions

Vector Customization Service

Account

License Types - Expanded

Expanded License EULA

1. VectorStock® grants you a Multi-seat, non-exclusive, non-transferable, license to use and reproduce vectors in the following ways, in addition to and including all Standard License applications.
   a. You may use the content on items (digital or hard copy) for resale such as t-shirts and fabric prints, flags, postcards, stickers, posters, coffee mugs, calendars or the like. Digital reproduction in non Vector formats (jpg, gif, bmp) on websites such as Cafepress or Digital Scrapbooking kits. You may use the content in commercial software applications or GUI design for resale with no limitations regarding the number of copies or print run.
   b. You may use the Image in website templates, document templates, wallpapers, screensavers, e-cards or similar products for resale with no limitations regarding the number of copies.

      All other restrictions from the "not permitted" section from the standard license still apply.
   c. All license agreements prohibit the resale or re distribution of any part or element of the content as a vector, or in any format that will allow a third party access to the file in digital vector format i.e EPS, Ai, Flash, SVG and the like.

      For example the content can not be resold or distributed in a Flash template unless the flash elements have been converted to a non-vector format.

   The EULA Standard or Expanded does not permit the use of any part of any purchased VectorStock® content on a competing website or business in any media format, or in any situation that may be regarded as a conflict of interest between VectorStock® and the end user and or their use of our content.

   Your Multi-seat license gives you the option to share the vector(s) with other people within your organization or company, not limited by the number of users within your organization or company. You warrant that you will do your best to prevent third parties from duplicating the content.

   Note: This license may be updated from time to time to include relevant new and future media usage/applications. This agreement is retrospective and supersedes any previous versions or modifications. All users will be notified of major changes via email.

VectorStock	Vector Images	Popular Categories	Social
Contact	102,152 Vectors Just Added	Background & Textures	twitter.com/vectorstock
FAQ	342,279 Free Vectors	Design elements	facebook.com/vectorstock
Become a Contributor	Most Popular Vectors	Icons & Emblems (sets)	pinterest.com/vectorstock
Affiliate	Buy Vector Art & Graphics	Floral & Decorative	youtube.com/vectorstock
What is a Vector?		Borders & Frames	

VectorStock Terms & Conditions

VECTORSTOCK and the VectorStock logo are registered trademarks of VectorStock Media. Copyright © 2020. All Rights Reserved.
